# Hepatic Encephalopathy Severity and Mortality Risk Stratification in Alcohol-Related Acute-on-Chronic Liver Failure

**DOI:** 10.3390/diagnostics16111741

**Published:** 2026-06-05

**Authors:** Tijana Glisic, Bojan Korica, Branko Beronja, Milica Djakovic, Nevena Baljosevic, Dusan Dj Popovic, Jelena Martinov Nestorov, Milica Stojkovic Lalosevic

**Affiliations:** 1 Clinic of Gastroenterology and Hepatology, University Clinical Center of Serbia, 11000 Belgrade, Serbia; koricabojan@gmail.com (B.K.); jelenamartinov@yahoo.com (J.M.N.); drmilicastojkovic@gmail.com (M.S.L.); 2 Faculty of Medicine, University of Belgrade, 11000 Belgrade, Serbia; djakovicmilica17@gmail.com (M.D.); dr.dusan.popovic@gmail.com (D.D.P.); 3 Clinic for Infectious and Tropical Diseases “Prof. Dr. Kosta Todorović”, 11000 Belgrade, Serbia; 4 Emergency Center, University Clinical Center of Serbia, 11000 Belgrade, Serbia; baljosevic.nevena@yahoo.com; 5 Department of Gastroenterology, Clinical and Hospital Center “Dr. Dragisa Misovic-Dedinje”, 11000 Belgrade, Serbia

**Keywords:** acute-on-chronic liver failure, hepatic encephalopathy, CLIF-C OF, APACHE II, SOFA

## Abstract

**Background/Objectives**: Acute-on-chronic liver failure (ACLF) is characterized by multiple organ failure and short-term mortality, and hepatic encephalopathy (HE) is its frequent complication. We investigated whether the severity of HE upon admission in patients with alcohol-related ACLF at the intensive care unit (ICU) was associated with short-term mortality. **Methods**: In total, 100 patients with alcohol-related ACLF and HE admitted in ICU were enrolled in the study. Laboratory biomarkers, total hospital length of stay (LOS), ICU length of stay, acute kidney injury (AKI), Acute Physiology and Chronic Health Evaluation II score, CLIF-C organ failure and Sequential Organ Failure Assessment score were tested in relation to the mortality risk. HE was assessed and divided into groups using the West Haven criteria. **Results**: Total hospital LOS, 7-day and 28-day mortality were significantly higher in the higher-grade HE group (*p* = 0.035, *p* = 0.031, *p* = 0.002, respectively). CLIF-C OF, SOFA, and APACHE II scores were significantly higher in the higher-grade HE group (*p* < 0.001). Kaplan–Meier survival analysis demonstrated reduced survival in patients with higher-grade HE (log-rank *p* < 0.001). In Cox regression analyses, AKI was associated with short-term mortality in both HE groups. Total hospital LOS and ICU length of stay were also associated with mortality, but were interpreted as post-baseline markers of clinical trajectory rather than baseline prognostic predictors. **Conclusions**: In patients with alcohol-related ACLF and HE, higher-grade HE was associated with poorer short-term survival. AKI and higher CLIF-C OF, SOFA and APACHE II scores were associated with poor outcomes, supporting their clinical relevance for mortality risk stratification in this population. LOS-related findings should be interpreted as markers of clinical trajectory rather than baseline prognostic predictors.

## 1. Introduction

Acute-on-chronic liver failure (ACLF) is a distinct clinical syndrome that occurs in the setting of chronic liver disease and is characterized by acute liver failure associated with organ dysfunction and high short-term mortality [1,2,3]. According to the number of organ failure (OF), ACLF is classified from grade 1 (less sever) to grade 3 (most sever). The high mortality observed in patients with ACLF treated in intensive care units (ICUs) is closely associated with multiple organ dysfunction [1]. It is important to emphasize that the type of organ(s), and not just the number of organs involved in ACLF, has a significant impact on mortality. Hepatic encephalopathy (HE) and renal failure, in particular, have been associated with high short-term mortality (28 days) [4,5]. Worldwide, the prevalence of ACLF is about 35% among those with decompensated cirrhosis, with the highest incidence in South Asia, estimated at 65%. The 90-day mortality rate can reach 58% to 68% in South Asia [6].

Hepatic encephalopathy (HE) is a frequent and challenging complication of chronic liver disease, particularly in patients with ACLF, with reported prevalence ranging from 17.2% to 57.8% [7,8]. HE is a major cause of ICU admission and is associated with high mortality in this population. Its multifactorial pathogenesis makes prediction and risk stratification difficult. HE severity is commonly assessed using the West Haven criteria, which classify patients according to changes in behavior and level of consciousness [9,10]. Several laboratory markers and prognostic models, including ammonia, MELD, MELD-Na and inflammatory indices, have been evaluated for HE prediction and outcome assessment [11,12,13,14]. In patients with ACLF, broader severity scores such as APACHE II and SOFA may be useful because they capture multiorgan dysfunction and critical illness severity [15,16,17]. Previous studies have shown that HE in ACLF is associated with increased mortality, particularly in patients with higher HE grades [18,19]. A significant factor contributing to the poor outcome of patients with ACLF is the development of infection. In these patients, infections are recognized as an independent risk factor for mortality, with rates reaching up to 70% within 30 days. Therefore, early recognition of infection and its prompt treatment are paramount for patient outcome [20,21,22]. The synergistic interplay of the systemic inflammation which exists in chronic liver disease and infection has been recognized as fundamental in the development and progression of hepatic encephalopathy. Proinflammatory cytokines activate cerebral endothelial cells and circulating immune cells can then adhere to the activated endothelium and be recruited by the brain parenchyma. As a result, activation of microglia and astrocyte occurs [23]. These immune cells then produce proinflammatory cytokines, propagating neuroinflammation and altering neurotransmission and behavior [24]. Additionally, in acute liver failure, neutrophils have reduced phagocytic activity, increasing susceptibility to bacterial infection [25,26].

The aim of our retrospective study was to evaluate whether the severity of hepatic encephalopathy upon admission in patients with ACLF at the intensive care unit is associated with in-hospital short-term mortality. Furthermore, we sought to identify the risk factors associated with severity of HE and short-term mortality.

## 2. Materials and Methods

### 2.1. Study Design and Patient Population

This retrospective cohort study included patients with acute-on-chronic liver failure (ACLF) admitted to the hepatology intensive care unit (ICU) of the Emergency Centre, University Clinical Centre of Serbia, a tertiary referral and the largest national center for the management of severe liver disease, between January 2020 and March 2025. A total of 100 patients with ACLF due exclusively to alcoholic liver disease were included. Patients were selected from the overall ACLF population treated at our institution during the study period, and only those with alcohol-related ACLF (“alcohol-on-alcohol ACLF”) were analyzed. The diagnosis of advanced chronic liver disease was based on relevant clinical findings, laboratory parameters, imaging methods including ultrasonography and/or computed tomography, and/or histological reports. All patients required ICU admission at presentation due to the severity of their condition, while a proportion of patients were subsequently transferred to standard hospital wards after clinical stabilization. Exclusion criteria included age below 18 years, presence of malignancy, prior liver or splenic surgery, previous transjugular intrahepatic portosystemic shunt placement, portal vein thrombosis, use of immunosuppressive therapy, and human immunodeficiency virus infection.

### 2.2. Definitions and Data Collection

The diagnosis of ACLF was established in accordance with the criteria of the European Association for the Study of the Liver–Chronic Liver Failure Consortium (EASL-CLIF) [1]. Organ failure was assessed using the CLIF-C organ failure (CLIF-C OF) score, which evaluates six organ systems including liver, kidney, brain, coagulation, circulation, and respiration, and ACLF severity was graded based on the number and type of organ failures [27]. Hepatic encephalopathy (HE) severity was graded at ICU admission according to the West Haven criteria [10] by an experienced gastroenterologist/hepatologist, based on bedside assessment of mental status, orientation, behavior, psychomotor function, neuromuscular signs and level of consciousness. Patients with West Haven grades I–II were categorized as lower-grade HE, whereas patients with grades III–IV were categorized as higher-grade HE. This classification represented the primary exposure variable for subsequent analyses.

Clinical and laboratory data were collected from electronic and written medical records at the time of ICU admission. Demographic and clinical variables included age, sex, presence of ascites, hepatic encephalopathy grade, history of prior liver-related hospitalizations, and comorbidities such as arterial hypertension, diabetes mellitus, cardiovascular diseases, chronic pulmonary diseases and chronic kidney diseases. Laboratory parameters included complete blood count with differential (red blood cells, hemoglobin, white blood cells, neutrophils, lymphocytes, monocytes, eosinophils, platelets), liver function tests (aspartate aminotransferase, alanine aminotransferase, alkaline phosphatase, gamma-glutamyl transferase, total bilirubin), coagulation parameters (international normalized ratio, fibrinogen), biochemical markers (albumin, blood urea nitrogen, creatinine, glucose, sodium, potassium, chlorides), and inflammatory markers including C-reactive protein and procalcitonin. The clinical manifestations of decompensated chronic liver disease identified in our patients were: ascites (tense or refractory), presence of esophageal varices, variceal bleeding, spontaneous bacterial peritonitis, acute kidney injury and jaundice. In addition, several prognostic scores were also calculated for each patient, including CLIF-C organ failure (CLIF-C OF) score [27], systemic immune-inflammation index (SII) [28], the model for end-stage liver disease (MELD) [29], model for end-stage liver disease—natrium (MELD-Na) [30], Sequential Organ Failure Assessment (SOFA) [17], Acute Physiology and Chronic Health Evaluation II (APACHE II) [16], Cirrhosis Acute Gastrointestinal Bleeding score (CAGIB) [31], and EVendo score [32].

Composite infection variables (e.g., total bloodstream infection and total urinary tract infection) were derived by grouping related infection subtypes.

All scores are presented in Table 1.

### 2.3. Outcome Measures and Statistical Analysis

The primary outcome of the study was 28-day mortality, defined as death occurring within 28 days from ICU admission. Time-to-event was calculated from the date of ICU admission to death within 28 days, while patients who survived beyond 28 days were censored at day 28. In addition, 7-day mortality was analyzed as a secondary short-term outcome.

Statistical analyses were performed using SPSS software (version 25.0, IBM, Chicago, IL, USA) and GraphPad Prism (version 8, GraphPad Software, San Diego, CA, USA). Continuous variables were expressed as mean ± standard deviation or median with interquartile range, depending on data distribution assessed using the Kolmogorov–Smirnov test, while categorical variables were presented as counts and percentages. Differences between groups defined by hepatic encephalopathy severity were evaluated using Student’s t-test or the Mann–Whitney U test for continuous variables and the chi-square test for categorical variables.

Survival analysis was performed using the Kaplan–Meier method, and differences between groups were assessed using the log-rank test. Time-to-event data were defined as the interval between ICU admission and death or censoring. Total hospital LOS and ICU LOS were considered post-baseline variables, as they were determined after ICU admission and during the follow-up period. Therefore, they were not interpreted as baseline prognostic predictors of 28-day mortality. When included in Cox regression analyses, LOS-related variables were analyzed as markers of clinical trajectory rather than causal or baseline predictors, given their susceptibility to reverse causality and their dependence on timing of death, treatment intensity and in-hospital complications.

To identify factors associated with mortality, Cox proportional hazards regression analyses were performed separately within the lower-grade and higher-grade hepatic encephalopathy cohorts. Univariable Cox regression analyses were initially conducted for all variables of interest. To account for the limited sample size and reduce the risk of overfitting, variables were organized into four predefined domains, and separate multivariable models were constructed for each domain rather than combining all variables into a single model. Collinearity among variables included in multivariable Cox models was assessed using variance inflation factors (VIFs) and tolerance statistics in linear regression models containing the same covariates. A VIF > 5 or tolerance < 0.20 was considered indicative of relevant collinearity.

Model 1 included clinical characteristics assessed at ICU admission, Model 2 comprised comorbidity-related variables, Model 3 included established prognostic severity scores, and Model 4 incorporated infection-related variables, including both infections at admission and hospital-acquired infections during ICU stay. Within each domain, variables that reached statistical significance in univariable analysis were entered into the corresponding multivariable model. Age and sex were retained in all models irrespective of statistical significance due to their established clinical relevance. This domain-based modeling strategy enabled a structured and clinically meaningful evaluation of factors associated with 28-day mortality, while limiting multicollinearity and preserving model stability. The proportional hazards assumption was assessed using graphical methods, including log-minus-log survival plots, and by evaluating Schoenfeld residuals where applicable. Results were expressed as hazard ratios with corresponding 95% confidence intervals, and a *p*-value less than 0.05 was considered statistically significant. Forest plots were generated using GraphPad Prism to visually present the results of multivariable Cox regression analyses.

### 2.4. Ethical Considerations

The study was approved by the Ethics Committee of the University Clinical Centre of Serbia (approval number: 772/8; approval date: 27 March 2025) and conducted in accordance with the Declaration of Helsinki. The analysis was retrospective and based on routinely collected medical record data extracted after ethics approval. Patient confidentiality was preserved by anonymization of the dataset before analysis. Informed consent procedures followed institutional regulations applicable to retrospective analyses of medical records.

## 3. Results

This study included 100 patients with alcohol-related acute-on-chronic liver failure (ACLF), stratified according to the severity of hepatic encephalopathy (HE) at hepatology ICU admission into lower-grade HE (West Haven I–II; *n* = 52) and higher-grade HE (West Haven III–IV; *n* = 48). All recorded deaths occurred within the predefined 28-day follow-up period. Patients who survived were censored on day 28.

Baseline characteristics, clinical features, and outcomes are presented in Table 2. There were no statistically significant differences between the groups in age or sex distribution. Total hospital length of stay was significantly shorter in the higher-grade HE group, while ICU length of stay did not differ between the groups. As LOS variables were determined after ICU admission, they were considered post-baseline clinical course variables rather than baseline prognostic factors. Seven-day and 28-day mortality were both significantly higher in the higher-grade HE cohort.

No statistically significant differences were observed between the groups in clinical manifestations of decompensated liver disease at ICU admission. The history of liver-related hospitalization was more frequent in the higher-grade HE group, without reaching statistical significance. Diabetes mellitus was more frequent in the lower-grade HE cohort, whereas other comorbidities did not differ significantly between groups.

Baseline laboratory parameters at ICU admission are presented in Supplementary Table S1.

Clinical severity scores at ICU admission are also shown in Table 2. CLIF-C OF, SOFA, and APACHE II scores were significantly higher in the higher-grade HE groups, whereas MELD and MELD-Na did not differ significantly. Overall, the higher-grade HE cohort showed a more severe acute clinical phenotype. Although MELD and MELD-Na did not differ significantly between groups, CLIF-C OF, SOFA and APACHE II were consistently higher in patients with higher-grade HE, suggesting that advanced HE in this ICU ACLF population was more closely aligned with global organ failure and critical illness severity than with liver-specific severity alone.

Infection-related characteristics are summarized in Table 3. At ICU admission, urinary tract infection and sepsis were the most frequent infectious presentations in both lower-grade and higher-grade HE cohorts (30.8% vs. 29.2% and 26.9% vs. 35.4%, respectively). During ICU stay, catheter-associated urinary tract infection was the most common hospital-acquired infection (26.9% vs. 25.0%), followed by primary bloodstream infection (17.3% vs. 22.9%) and pneumonia-related complications. Although no significant between-group differences were observed, these data indicate a high infectious burden in both cohorts. Further cross-stratification was not performed because patients could have multiple infections and the number of events within individual subtypes was limited.

**Figure 1:**
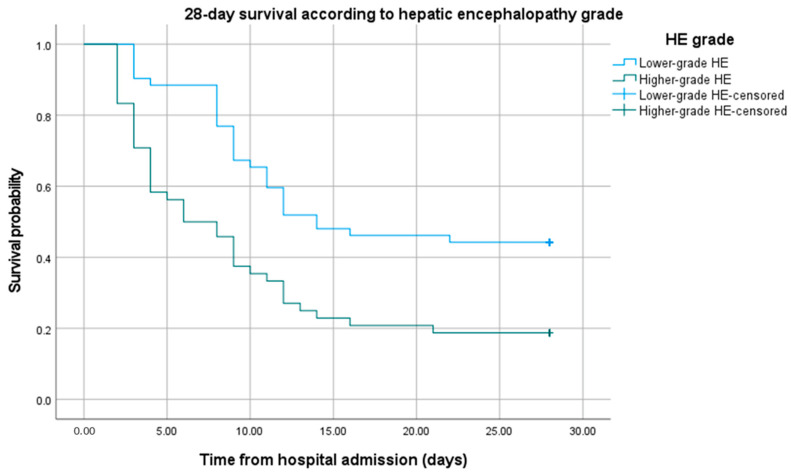
Kaplan–Meier survival curves according to hepatic encephalopathy severity (lower-grade vs. higher-grade HE) in patients with alcohol-related ACLF.

Kaplan–Meier survival analysis demonstrated a significant difference between the groups, with reduced survival in patients with higher-grade HE (log-rank *p* < 0.001; Figure 1). Higher-grade HE was associated with mortality, corresponding to a 134% higher hazard of death compared with lower-grade HE (HR 2.34, 95% CI 1.15–2.98, *p* = 0.007).

### Predictors of 28-Day Mortality in Lower-Grade and Higher-Grade HE Cohorts

In the lower-grade HE cohort, AKI was associated with 28-day mortality in multivariable analysis. Total hospital LOS also showed an association with mortality; however, given its post-baseline nature, this finding was interpreted as a marker of clinical trajectory rather than a baseline prognostic predictor. In the higher-grade HE cohort, acute kidney injury remained associated with 28-day mortality in multivariable analysis, with more than a twofold increase in mortality risk. Total hospital LOS and ICU LOS were also associated with mortality; each one-day increase in total hospital LOS corresponded to an approximately 96% higher hazard of death, while each additional ICU day corresponded to an approximately 94% higher hazard. Given that both LOS variables were determined during hospitalization, these associations were interpreted cautiously and considered to reflect the subsequent clinical course rather than prognostic information available at ICU admission. Analysis of comorbidity-related variables did not identify independent predictors of mortality in multivariable models. Collinearity diagnostics did not indicate relevant collinearity among variables included in the multivariable Cox regression models, with all VIF values below 5 and tolerance values above 0.20.

Clinical characteristics and comorbidity-related factors associated with 28-day mortality in lower-grade and higher-grade HE cohorts are shown in Table 4.

Analysis of prognostic scores (Table 5) demonstrated that MELD, CLIF-C OF, SOFA, and APACHE II scores were independently associated with mortality in both cohorts. In the lower-grade HE cohort, each unit increase in MELD score was associated with an approximately 3% increase in mortality risk, CLIF-C OF with a 17% increase, SOFA with a 13% increase, and APACHE II with a 7% increase. In the higher-grade HE cohort, each unit increase in MELD score was associated with an approximately 4% increase in mortality risk, CLIF-C OF with a 21% increase, SOFA with a 16% increase, and APACHE II with an 8% increase.

Infection-related variables were associated with mortality in univariable analyses in both cohorts. In multivariable analysis, only sepsis at admission remained independently associated with mortality in the higher-grade HE cohort, corresponding to an approximately 87% higher hazard of death.

**Figure 2:**
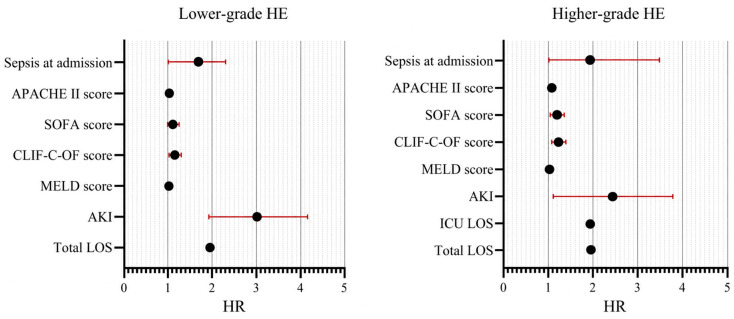
Forest plot of multivariable Cox proportional hazards regression models for 28-day mortality in lower-grade and higher-grade hepatic encephalopathy cohorts. Hazard ratios (HR) with 95% confidence intervals (CIs) are presented for independent predictors identified in each model.

The forest plot of multivariable Cox regression models is shown in Figure 2. AKI was associated with mortality in both HE severity cohorts. Total hospital LOS and ICU LOS are shown in the figure because of their association with mortality, but their interpretation requires caution because they were determined during hospitalization and may reflect the subsequent clinical course rather than risk at ICU admission. Prognostic scores remained associated with mortality in both groups, while sepsis at admission was associated with mortality only in the higher-grade HE cohort. Based on the forest plot, AKI showed the strongest clinical association with mortality in both HE severity cohorts. Among prognostic scores, CLIF-C OF had the highest per-point hazard estimate, followed by SOFA, APACHE II and MELD. However, these estimates should be compared cautiously because the scores differ in scale, composition and clinical meaning. In the higher-grade HE cohort, sepsis at admission represented the main infection-related factor associated with mortality.

## 4. Discussion

HE is one of the life-threatening complications that occurs in patients with chronic liver disease. Patients with ACLF are at high risk of mortality due to impaired liver function leading to multi-organ failure. When ACLF is complicated by HE, the risk of poor outcome further increases, highlighting early recognition of this condition and prompt initiation of treatment.

In a subpopulation of patients with alcohol-related chronic liver disease who had experienced ACLF, we investigated how the severity of HE affects short-term mortality of patients admitted to the ICU, as well as whether there are differences in clinical characteristics, values of non-invasive prognostic scores, and the presence of infection or sepsis in these groups of patients.

In our study, the age and gender distribution within lower-grade and higher-grade HE cohorts of ACLF did not differ significantly. This finding is consistent with Peng et al. where ages and gender presented no significant differences between two groups [33]. Patients with higher-grade HE experienced reduced total length of hospitalization, likely reflecting a more rapid fatal outcome compared to the group with lower-grade HE. In addition, 7-day and 28-day mortality rate was significantly higher in the higher-grade HE group. Although not statistically significant, a previous history of hospitalization in patients with higher-grade HE was more frequent. Ballester et al. reported that a previous history of HE was associated with an increased risk of hospitalization due to OHE in patients with ACFL [34]. Similar, a study by Maggi DC et al. researched factors associated with absence of improvement HE in patients with acute decompensation of liver cirrhosis and showed that previous HE and ACLF were independently associated with a progression of mental status in this setting [35]. There are also certain specificities for ACLF-associated HE. This kind of HE is associated with an excessive generalized inflammatory response that may play a significant role in brain dysfunction. There is increasing suggestion that ACLF-related HE can be distinguished as a separate entity from isolated HE [20]. This inflammatory component has direct clinical relevance. In ACLF, proinflammatory cytokines may amplify ammonia-related astrocyte dysfunction, promote microglial activation and alter neurotransmission, contributing to progressive impairment of cognition, behavior, psychomotor function and consciousness [23,24,25]. HE shows neuropsychiatric symptoms by increasing brain edema and intracranial hypertension [36]. In HE, ammonia crosses the blood–brain barrier and ammonia uptake is increased in the brain [37]. This hyperammonemia condition induces astrocyte swelling and brain edema [38]. HE may be characterized by systemic inflammation, which can influence cerebral inflammation [39]. Microglia control the immune and inflammatory responses in the central nervous system [40], and liver disease can influence the functional change and polarization of microglia in the brain [41,42]. Some previous studies have shown that in HE microglial activation produces various inflammatory cytokines [43]. Circulating factors, such as pro-inflammatory chemokines, including interleukin-6 (IL-6), interleukin-1β (IL-1β), tumor necrosis factor-α (TNF-α), and chemokine monocyte chemoattractant protein-1, can cause blood–brain barrier breakdown, and can be accompanied by astrocyte swelling and glial activation which induce neuroinflammation, which is responsible for neuronal cell damage in HE. All these events lead to cognitive and motor dysfunction [44]. The levels of circulating ammonia and pro-inflammatory cytokines, such as IL-6 and IL-18, affect the severity of cognitive dysfunction, and the interplay between hyperammonaemia and inflammation is involved in cognitive deterioration in HE patients [45]. Thus, higher-grade HE may reflect the convergence of liver failure, systemic inflammation and extrahepatic organ dysfunction, rather than neurological impairment alone, which is consistent with its association with poorer short-term survival in our cohort.

Among comorbidities, diabetes mellitus was the only variable that differed significantly between the HE severity groups, but it was more frequent in the lower-grade HE cohort. This finding is not in line with previous studies suggesting an association between diabetes and HE in cirrhosis [46,47,48,49,50]. Proposed mechanisms include autonomic dysfunction with impaired intestinal motility, constipation and small intestinal bacterial overgrowth (SIBO), leading to increased ammonia production, as well as systemic inflammation and greater susceptibility to infections [46,47,48,49,50,51]. However, this observation should be interpreted cautiously because of the small number of patients with diabetes in our cohort. In ICU-admitted patients with alcohol-related ACLF, HE severity may be driven predominantly by acute organ failure, systemic inflammation and precipitating events rather than by baseline metabolic comorbidity. Investigation of the predictive performances of non-invasive models between a group of patients with lower-grade HE and higher-grade HE with ACLF, CLIF-C OF, SOFA and APACHE II scores showed significantly higher values in the higher-grade HE group. Our results agree with the findings of other studies [52]. Mikolasevic et al. identified APACHE II score as the best predictor of short-term mortality [53], while Garg et al. highlighted SOFA and APACHE II as the most reliable for predicting 28-day outcome in patients with ACLF [54]. CANONIC study data demonstrated that CLIF-C OF was able to defeat MELD and MELD-Na scores when predicting short-term mortality in patients with ACLF [1,55]. Kim et al. in their study revealed that the CLIF-C ACLF is superior for predicting 28-day mortality in alcohol-related ACLF than MELD and MELD-Na [56]. The variability in the prognostic accuracy among different non-invasive scores in ACLF is likely because ACLF is an entity per se with high mortality, which may be independent of the severity scores. Considering that the MELD score had shown the greatest predictive ability in predicting mortality in patients with end-stage chronic liver disease, our results are not in accordance with findings of some other studies [57]. The explanation could be that the MELD score was not created for patients with ACLF because this condition has special clinical and pathophysiological characteristics.

Infections, particularly bacterial infections, are a trigger for systemic inflammation and ACLF in patients with chronic liver disease [58]. Various mechanisms lead to increased inflammation in these patients, increased oxidative stress, and the development of ACLF [59]. In alcohol-related ACLF, bacterial infections associated with alcohol-related hepatitis cause further increase in systemic inflammation and worsening survival [60]. Our study did not show differences in the presence of infections during ICU hospitalization between the observed groups, which can be explained by the relatively small sample size and the overall severity of illness in our cohort of patients.

In the present study we analyzed short-term mortality in the observed cohorts and found reduced survival in patients with higher-grade HE. The poorer survival observed in patients with higher-grade HE is biologically plausible. In ACLF, HE severity probably reflects the convergence of liver-related neurotoxicity, systemic inflammation and extrahepatic organ failure. Ammonia-mediated astrocyte dysfunction may be amplified by systemic inflammation and immune dysregulation, promoting neuroinflammation and altered neurotransmission [20,23,24,25]. Clinically, this may translate into progressive impairment of cognition, behavior, psychomotor function and level of consciousness, corresponding to the transition from lower-grade to higher-grade HE. Thus, higher-grade HE should be interpreted not only as a neurological complication, but also as a marker of advanced systemic disease in ACLF. Maggi et al. showed that a higher proportion of patients who experienced adverse HE progression was among patients with ACLF at admission in ICU [35]. We must highlight that we could not find previous studies that evaluated factors associated with short mortality among subgroups of patients with HE as we divided them into lower-grade and higher-grade HE, so we did not have adequate data for comparison.

Our data showed an independent association of total length of hospitalization and AKI with mortality in both examined groups. Renal failure is the most frequent organ failure in ACLF and is ruled by high-grade inflammation [61]. Cordoba et al. demonstrated that renal and brain failure are independent predictors of mortality, and in the CANONIC study, the kidney was the most affected organ in patients with ACLF [20,62]. Fisher et al. also showed that patients with cirrhosis and AKI face very high short-term mortality and are at substantial risk of ACLF development and progression [63]. These findings support the results of our investigation, where patient’s prognosis is negatively affected by the presence of both renal and brain failure and associated with short-term mortality. However, the association between AKI and mortality may also reflect overall ACLF severity rather than an isolated effect of renal dysfunction. In critically ill patients, AKI frequently occurs together with circulatory failure, infection, systemic inflammation and greater need for organ support, which may contribute to residual confounding despite multivariable adjustment. There is less data on the total length of hospitalization and ICU length of stay as factors associated with increased mortality in patients with ACLF-related HE. Our finding indicate that these factors are notably associated with mortality in patients with both lower and higher-grade HE. The study by Soares et al. found that extending intensive care support to seven days may improve outcomes in patients with multiple OF within ACLF [64]. Unfortunately, our results did not show a benefit of ICU length of stay towards a better outcome. There are many potential reasons for such a result, like more frequent development of nosocomial infections, more frequent need for mechanical ventilator support and frequent complications, as well as a more severe form of the disease.

In both of our investigated ACLF-related HE groups, MELD, CLIF-C OF, SOFA and APACHE II scores demonstrated independent association with short-term mortality. These findings are consistent with some other studies. Saxena et al. pointed out that the MELD score is the most reliable for predicting 28-day mortality [65]. A study performed by Perdigoto et al. showed that the CLIF-C OF score has good accuracy while MELD score was superior to other predictive scores for 90-day mortality [66]. Taş et al. found that APACHE II showed better discriminative value of prognosis for cirrhotic patients with HE who were admitted in ICU than MELD [52]. Analyzing how much of an increase in each score unit affects the increase in the mortality rate, the above scores can indicate with great reliability their predictive value in patients with ACLF-related hepatic encephalopathy. All scores use a significant number of parameters which can explain their role and the result they provide. Based on the results of the CANONIC study, two conclusions emerge. The first one is that ACLF-related HE occurs more frequently in younger individuals, in the presence of bacterial infection, active alcoholism, and dilutional hyponatremia. The second conclusion is that an excessive generalized inflammatory response may play a role in brain dysfunction [20]. In addition, another study has shown a clear correlation between systemic inflammation and advanced HE, leading to an increased risk of mortality [67].

Infections could trigger and worsen the severity and prognosis of HE. We only focused on admission infections to ensure consistency in our study, and results showed that infection-related variables were associated with mortality. When analyzing a presence of cognitive impairment, Merli et al. confirmed it in 79% of patients with infections and in 90% with sepsis [68]. Previous clinical evidence has demonstrated the impact of infections on the mortality rate of cirrhotic patients with HE. Their study showed that pneumonia and sepsis without specific focus were particularly harmful to these patients [69].

Immune dysregulation contributes to the pathogenesis of ACLF and increases the risk of infection. Our study was conducted on patients with alcohol-related ACLF and HE. The specificity of alcoholic chronic liver disease with the development of ACLF is that chronic alcohol use leads to immune dysfunction, gastrointestinal dysbiosis and increased intestinal permeability. As a result, bacterial translocation and chronic persistent inflammation occur. All this contributes to the easier occurrence of infections and the development of sepsis [70,71]. In our study, patients with sepsis-related ACLF on admission to the ICU who had higher-grade HE had significantly higher mortality compared to the lower-grade HE group, and sepsis on admission was shown to be an independently associated factor with mortality in the studied group of patients with higher-grade HE. von Maldeghem et al. presented results in which the clinical consequences in patients with sepsis-related ACLF were extremely significant. These patients more often presented with higher-grade ACLF and required more intensive supportive therapy (vasopressor therapy, respiratory support, renal replacement therapy) that was associated with a hospital mortality of 60% [72]. The interpretation of infection-related findings is also limited by treatment intensity and timing. Infections and sepsis may directly worsen ACLF and HE, but they may also identify patients with more severe disease, longer ICU exposure, invasive procedures, mechanical ventilation or vasopressor requirement. Therefore, these associations should be interpreted as clinically meaningful but not necessarily causal.

This study has several limitations. First, its retrospective, single-center design and limited sample size restrict causal inference and generalizability. Second, residual confounding cannot be excluded, particularly because treatment intensity and ICU-level interventions were not fully captured in the analysis. Variables such as mechanical ventilation, vasopressor use, renal replacement therapy, sedation or intubation status, and HE-directed treatment may have influenced both HE grading and mortality. Third, some variables, including total hospital LOS, ICU LOS and hospital-acquired infections, were determined after ICU admission and may therefore be influenced by reverse causality, survival time and in-hospital complications. Finally, because data were collected from medical records, misclassification and incomplete documentation cannot be fully excluded.

## 5. Conclusions

In this retrospective analysis of 100 ICU-admitted patients with alcohol-related ACLF and HE, higher-grade HE was associated with poorer short-term survival. CLIF-C OF, SOFA, APACHE II and MELD scores, as well as AKI, were associated with short-term mortality in both HE severity cohorts, while sepsis-triggered ACLF was associated with mortality in the higher-grade HE subgroup. Hospital LOS and ICU LOS were associated with mortality but should be interpreted as post-baseline markers of clinical trajectory rather than baseline prognostic predictors. These findings emphasize the importance of early recognition and intensive management of HE, renal dysfunction, infection and sepsis in patients with alcohol-related ACLF.

## Figures and Tables

**Table 1:** Formula for calculations of non-invasive scores.

MELD score = 9.57_ln(Cr) + 3.78_ln(TBIL) + 11.2_ln(INR) + 6.43
MELD-Na = MELD + 1.32 × (137-Na) − [0.033 × MELD × (137-Na)]
S II = (Neu × Plt)/Ly
CAGIB = (Diabetes (yes = 1, no = 0) × 1.040) + (HCC (yes = 1, no = 0) × 0.974) + (TBIL × 0.005) − (ALB × 0.091) + (ALT × 0.001) + (Cr × 0.012) − 3.964.
CLIF-C ACLF = 10 × [0.33 × CLIF-C OF + 0.04 × age + 0.63 × ln(WBC) − 2]

**Table 2:** Baseline characteristics, clinical features, and outcomes of patients with lower-grade and higher-grade hepatic encephalopathy at hepatology ICU admission.

Variable		Lower-Grade HE *N* = 52	Higher-Grade HE *N* = 48	*p*
Sex	Male	40 (76.9%)	37 (77.1%)	0.587
	Female	12 (23.1%)	11 (22.9%)	
Age		58.0 ± 13.31	56.65 ± 12.56	0.503
Total length of hospital stays (days)		12.0 (8.5–15.5)	9.0 (4.0–16.0)	**0.035**
ICU length of stay (days)		8.0 (4.0–12.0)	8.0 (4.0–12.0)	>0.999
7-day mortality		15 (28.8%)	20 (41.7%)	**0.031**
28-day mortality		29 (55.7%)	39 (81.2%)	**0.002**
History of liver-related hospitalization		18 (34.6%)	26 (54.2%)	0.069
**Clinical manifestations of decompensated liver disease at hepatology ICU admission**				
Ascites		45 (86.5%)	38 (79.2%)	0.426
Tense or refractory ascites		14 (26.9%)	11 (22.9%)	0.818
Variceal bleeding		5 (9.6%)	10 (20.8%)	0.162
Esophageal varices		25 (48.1%)	24 (50.0%)	0.987
Spontaneous bacterial peritonitis		6 (11.5%)	8 (16.7%)	0.568
Acute kidney injury		8 (15.4%)	11 (22.9%)	0.445
Jaundice		36 (69.2%)	28 (58.3%)	0.300
**Comorbidities**				
Chronic heart failure		11 (21.2%)	8 (16.7%)	0.618
Arterial hypertension		14 (26.9%)	12 (25.0%)	0.952
Diabetes mellitus		9 (17.3%)	4 (8.3%)	**0.039**
Chronic kidney disease		4 (7.7%)	3 (6.2%)	0.915
COPD		2 (3.8%)	2 (4.2%)	>0.999
Cerebrovascular disease		1 (1.9%)	2 (4.2%)	0.606
AI and RE diseases		1 (1.9%)	2 (4.2%)	0.606
**Clinical severity and prognostic scores at ICU admission**				
MELD score, med (IQR)		29.00 (23.75–36.25)	25.00 (21.00–30.00)	0.070
MELD-Na score, med (IQR)		30.00 (24.75–36.25)	29.00 (21.50–31.00)	0.167
CLIF-C-OF score, med (IQR)		11.00 (10.00–11.25)	13.00 (11.00–14.25)	**<0.001**
SOFA score, med (IQR)		9.00 (7.00–10.25)	12.00 (10.00–14.00)	**<0.001**
APACHE II score, med (IQR)		18.00 (14.50–22.50)	26.50 (23.00–31.00)	**<0.001**
EVendo score, med (IQR)		7.14 (4.53–13.46)	6.94 (5.08–11.07)	0.619
SII, med (IQR)		1758.5 (604.5–3946.0)	2329.0 (1659.5–3001.5)	0.513
CAGIB score, med (IQR)		−6.93 (−8.58–−5.06)	−7.67 (−8.76–−6.37)	0.163

**Legend:** COPD—Chronic Obstructive Pulmonary Disease; MELD—Model for End-Stage Liver Disease; MELD-Na—Model for End-Stage Liver Disease with serum sodium; CLIF-C-OF—Chronic Liver Failure Consortium Organ Failure Score; SOFA—Sequential Organ Failure Assessment Score; APACHE II—Acute Physiology and Chronic Health Evaluation II; EVendo—Esophageal Varices Endoscopic Score; SII—Systemic Immune-Inflammation Index; CAGIB—Chinese Acute Gastrointestinal Bleeding Score. Statistically significant differences are shown in bold.

**Table 3:** Infections at hepatology ICU admission and hospital-acquired infections during ICU stay in patients with lower-grade and higher-grade hepatic encephalopathy.

Variable	Lower-Grade HE *N* = 52	Higher-Grade HE *N* = 48	*p*
**Infections at hepatology ICU admission**			
Sepsis	14 (26.9%)	17 (35.4%)	0.659
Respiratory infection	9 (17.3%)	10 (20.8%)	0.816
Urinary tract infection	16 (30.8%)	14 (29.2%)	0.641
Skin and soft tissue infection	4 (7.7%)	2 (4.2%)	0.422
**Hospital-acquired infections during hepatology ICU stay**			
Primary bloodstream infection	9 (17.3%)	11 (22.9%)	0.801
Catheter-related bloodstream infection	4 (7.7%)	5 (10.4%)	0.989
Secondary bloodstream infection	3 (5.8%)	3 (6.3%)	0.997
Ventilator-associated pneumonia	5 (9.6%)	8 (16.7%)	0.549
Hospital-acquired pneumonia	6 (11.5%)	7 (14.6%)	0.872
Catheter-associated urinary tract infection	14 (26.9%)	12 (25.0%)	0.634
Other urinary tract infection	2 (3.8%)	2 (4.2%)	0.912
Wound site infection	4 (7.7%)	2 (4.2%)	0.422

**Legend:** ICU—Intensive care unit. Patients may have more than one infection.

**Table 4:** Clinical characteristics and comorbidity-related factors associated with 28-day mortality in lower-grade and higher-grade hepatic encephalopathy cohorts: results of Cox proportional hazards regression models (time from hospital admission to outcome, in days).

	Lower-Grade HE						Higher-Grade HE					
Model 1	Univariate			Multivariate			Univariate			Multivariate		
	HR	95% CI	*p*	HR	95% CI	*p*	HR	95% CI	*p*	HR	95% CI	*p*
Age	1.01	0.98–1.04	0.410				1.02	0.98–1.06	0.290			
Sex (male)	0.92	0.45–1.88	0.810				1.64	0.79–3.40	0.190			
Total LOS (days)	1.96	1.92–2.00	**0.018**	1.97	1.93–2.01	**0.034**	1.95	1.91–1.99	**0.018**	1.96	1.92–2.00	**0.049**
ICU LOS (days)	1.95	0.90–3.01	0.110				1.93	1.88–1.98	**0.010**	1.94	1.89–1.99	**0.027**
Ascites	0.63	0.28–0.94	**0.042**	0.71	0.30–1.70	0.450	0.81	0.33–2.02	0.660			
Variceal bleeding	0.74	0.26–2.08	0.570				1.72	0.70–4.23	0.230			
EV	0.88	0.45–1.74	0.710				0.94	0.46–1.93	0.870			
SBP	1.39	0.55–3.53	0.480				1.58	0.62–4.03	0.340			
AKI	2.18	1.01–4.72	**0.047**	3.04	1.93–4.49	**0.044**	2.76	1.31–5.82	**0.007**	2.43	1.11–3.75	**0.026**
Jaundice	1.34	0.68–2.63	0.390				1.11	0.54–2.28	0.770			
**Model 2**	**Univariate**			**Multivariate**			**Univariate**			**Multivariate**		
	**HR**	**95% CI**	***p***	**HR**	**95% CI**	***p***	**HR**	**95% CI**	***p***	**HR**	**95% CI**	***p***
Age	1.01	0.98–1.04	0.410	1.00	0.96–1.05	0.920	1.02	0.98–1.06	0.290	1.02	0.97–1.07	0.420
Sex (male)	0.92	0.45–1.88	0.810	0.78	0.33–1.86	0.570	1.64	0.79–3.40	0.190	1.55	0.68–3.56	0.300
CHF	1.44	0.59–3.52	0.420	1.31	0.48–3.57	0.600	1.88	0.64–5.55	0.250	1.63	0.53–5.04	0.390
HTA	1.07	0.45–2.53	0.880	0.95	0.36–2.52	0.920	1.94	0.71–5.30	0.200	1.72	0.59–4.99	0.320
DM	1.21	0.46–3.20	0.700	1.14	0.39–3.31	0.810	1.12	0.32–3.87	0.860	0.97	0.26–3.62	0.960
CKD	1.58	0.43–5.78	0.490	1.41	0.34–5.81	0.640	1.73	0.36–8.25	0.490	1.50	0.29–7.71	0.630
COPD	1.02	0.14–7.35	0.990	0.86	0.10–7.25	0.890	0.64	0.08–5.06	0.670	0.52	0.06–4.42	0.550
CVI	1.74	0.19–15.80	0.620	1.55	0.15–15.70	0.710	2.02	0.29–14.00	0.480	1.78	0.23–13.80	0.580
AI and RE diseases	0.73	0.09–5.93	0.770	0.66	0.07–6.32	0.720	1.21	0.17–8.61	0.850	1.08	0.14–8.36	0.940
CCI	1.28	0.95–1.72	0.100	1.21	0.88–1.66	0.240	1.39	1.02–1.89	**0.036**	1.31	0.94–1.82	0.110

Legend: CI—Confidence Interval; HR—Hazard Ratio; LOS—Length of Stay; ICU—Intensive Care Unit; EV—Esophageal Varices; SBP—Spontaneous Bacterial Peritonitis; AKI—Acute Kidney Injury; CHF—Chronic Heart Failure; HTA—Arterial Hypertension; DM—Diabetes Mellitus; CKD—Chronic Kidney Disease; COPD—Chronic Obstructive Pulmonary Disease; CVI—Cerebrovascular Disease; AI—Autoimmune Diseases; RE—Rheumatic Diseases; CCI—Charlson Comorbidity Index. Statistically significant differences are shown in bold. Note: Total hospital LOS and ICU LOS are post-baseline variables determined after ICU admission. Their associations with mortality should be interpreted as markers of clinical course rather than baseline prognostic predictors because of potential reverse causality.

**Table 5:** Disease severity scores and infection-related factors associated with 28-day mortality in lower-grade and higher-grade hepatic encephalopathy cohorts: results of Cox proportional hazards regression models (time from hospital admission to outcome, in days).

	Lower-Grade HE						Higher-Grade HE					
Model 3	Univariate			Multivariate			Univariate			Multivariate		
	HR	95% CI	*p*	HR	95% CI	*p*	HR	95% CI	*p*	HR	95% CI	*p*
Age	1.01	0.98–1.04	0.410				1.02	0.98–1.06	0.290			
Sex (male)	0.92	0.45–1.88	0.810				1.64	0.79–3.40	0.190			
MELD score	1.05	1.02–1.08	**0.001**	1.03	1.00–1.07	**0.041**	1.06	1.03–1.09	**<0.001**	1.04	1.01–1.08	**0.017**
MELD-Na score	1.04	1.01–1.07	**0.006**	1.02	0.99–1.06	0.120	1.05	1.02–1.08	**0.002**	1.03	1.00–1.07	0.064
CLIF-C-OF score	1.22	1.10–1.35	**<0.001**	1.17	1.05–1.31	**0.005**	1.28	1.16–1.42	**<0.001**	1.21	1.08–1.36	**0.001**
SOFA score	1.18	1.08–1.30	**<0.001**	1.13	1.02–1.26	**0.020**	1.21	1.10–1.34	**<0.001**	1.16	1.04–1.30	**0.010**
APACHE II score	1.09	1.04–1.14	**<0.001**	1.07	1.01–1.13	**0.018**	1.11	1.05–1.16	**<0.001**	1.08	1.02–1.14	**0.011**
EVendo score	1.03	0.97–1.10	0.318				1.04	0.97–1.12	0.272			
SII	1.02	0.99–1.05	0.209				1.03	0.99–1.06	0.152			
CAGIB score	0.97	0.92–1.03	0.288				0.95	0.90–1.01	0.091			
**Model 4**	**Univariate**			**Multivariate**			**Univariate**			**Multivariate**		
	**HR**	**95% CI**	***p***	**HR**	**95% CI**	***p***	**HR**	**95% CI**	***p***	**HR**	**95% CI**	***p***
Age	1.01	0.98–1.04	0.410				1.02	0.98–1.06	0.290			
Sex (male)	0.92	0.45–1.88	0.810				1.64	0.79–3.40	0.190			
Infections at admission	1.61	0.93–2.78	0.088				1.84	1.04–3.26	**0.037**	1.62	0.89–2.95	0.110
Sepsis at admission	1.92	1.05–3.51	**0.034**	1.71	1.02–2.32	0.103	2.11	1.17–3.79	**0.013**	1.87	1.01–3.46	**0.047**
HAI	1.58	0.88–2.83	0.122				1.72	0.96–3.09	0.067			
Total bloodstream infection	1.83	1.02–3.30	**0.043**	1.62	0.87–3.02	0.129	2.07	1.14–3.75	**0.017**	1.84	0.98–3.45	0.076
Total UT infection	1.47	0.81–2.67	0.204				1.65	0.90–3.02	0.105			
VAP	1.76	1.04–3.30	**0.037**	1.55	0.80–3.02	0.191	2.03	1.11–3.71	**0.022**	1.81	0.96–3.42	0.096
HAP	1.64	0.88–3.06	0.119				1.89	1.03–3.46	**0.040**	1.66	0.87–3.18	0.125

**Legend:** HR—Hazard Ratio; CI—Confidence Interval; MELD—Model for End-Stage Liver Disease; MELD-Na—Model for End-Stage Liver Disease with serum sodium; CLIF-C-OF—Chronic Liver Failure Consortium Organ Failure Score; SOFA—Sequential Organ Failure Assessment Score; APACHE II—Acute Physiology and Chronic Health Evaluation II; EVendo—Esophageal Varices Endoscopic Score; SII—Systemic Immune-Inflammation Index; CAGIB—Chinese Acute Gastrointestinal Bleeding Score; HAI—Hospital-Acquired Infections; UT—Urinary Tract; VAP—Ventilator-Associated Pneumonia; HAP—Hospital-Acquired Pneumonia. Statistically significant differences are shown in bold.

## Data Availability

The raw data supporting the conclusions of this article will be made available by the authors on request.

## Supplementary Materials

The following supporting information can be downloaded at: https://www.mdpi.com/article/10.3390/diagnostics16111741/s1, Table S1. Laboratory parameters at ICU admission according to hepatic encephalopathy severity.
