# Supplementary material for: Hepatic Encephalopathy Severity and Mortality Risk Stratification in Alcohol-Related Acute-on-Chronic Liver Failure
Source: Diagnostics (Basel). 2026 Jun 5;16(11):1741. doi: 10.3390/diagnostics16111741 (PMC13256075; doi:10.3390/diagnostics16111741)
Supplement: Supplementary file 1 [file diagnostics-16-01741-s001.zip › diagnostics-4322273-supplementary.pdf]

Supplementary Table S1. Laboratory parameters at ICU admission according to hepatic encephalopathy severity

Variable	Lower-grade HE, med (IQR)	Higher-grade HE, med (IQR)	p
Hgb (g/L)	103.0 (88.0–119.0)	96.0 (82.0–111.0)	0.184
WBC (10^9/L)	10.8 (7.2–15.6)	14.2 (9.8–20.4)	**0.031**
Neutrophils (10^9/L)	8.6 (5.4–12.9)	11.9 (8.1–17.3)	**0.018**
Lymphocytes (10^9/L)	0.82 (0.48–1.24)	0.61 (0.35–0.96)	0.092
Monocytes (10^9/L)	0.74 (0.42–1.08)	0.81 (0.51–1.21)	0.427
Eosinophils (10^9/L)	0.04 (0.01–0.11)	0.02 (0.00–0.07)	0.156
Platelets (10^9/L)	91.0 (56.0–138.0)	76.0 (43.0–119.0)	0.238
TBIL (μmol/L)	312.0 (187.0–463.0)	286.0 (164.0–421.0)	0.412
Alb (g/L)	28.0 (24.0–32.0)	25.0 (21.0–29.0)	**0.046**
AST (U/L)	118.0 (72.0–214.0)	132.0 (81.0–236.0)	0.563
ALT (U/L)	61.0 (38.0–104.0)	69.0 (41.0–118.0)	0.484
ALP (U/L)	142.0 (101.0–211.0)	158.0 (112.0–236.0)	0.394
GGT (U/L)	96.0 (54.0–186.0)	104.0 (61.0–198.0)	0.617
sCr (μmol/L)	109.0 (82.0–164.0)	171.0 (112.0–253.0)	**0.006**
BUN (mmol/L)	10.8 (7.1–17.4)	17.2 (11.3–25.6)	**0.004**
LDH (U/L)	346.0 (248.0–512.0)	489.0 (326.0–731.0)	**0.021**
CRP (mg/L)	68.0 (34.0–124.0)	112.0 (59.0–181.0)	**0.014**
Pct (ng/mL)	0.72 (0.28–2.14)	1.86 (0.64–5.72)	**0.009**
Na (mmol/L)	134.0 (130.0–138.0)	130.0 (126.0–135.0)	**0.032**
K (mmol/L)	4.1 (3.6–4.7)	4.4 (3.8–5.1)	0.173
Cl (mmol/L)	101.0 (96.0–106.0)	98.0 (93.0–103.0)	0.087
INR	1.72 (1.38–2.31)	2.04 (1.56–2.78)	0.058

Legend: HE — hepatic encephalopathy; Hgb — hemoglobin; WBC — white blood cell count; TBIL — total bilirubin; Alb — albumin; AST — aspartate aminotransferase; ALT — alanine aminotransferase; ALP — alkaline phosphatase; GGT — gamma-glutamyl transferase; sCr — serum creatinine; BUN — blood urea nitrogen; LDH — lactate dehydrogenase; CRP — C-reactive protein; Pct — procalcitonin; Na — sodium; K — potassium; Cl — chloride; INR — international normalized ratio; IQR — interquartile range. Statistically significant differences are shown in **bold**.
